# Using the Relative Pitch Size, Internal Floaters, and External Floaters During Different Small-Sided Games in Professional Soccer in the Return-to-Play Process

**DOI:** 10.3390/jfmk11030327

**Published:** 2026-08-23

**Authors:** Michael C. Rumpf, Johannes Jäger, João Renato Silva, Matthias Lochmann

**Affiliations:** 1 Department of Sport Science and Sport, Friedrich-Alexander University Erlangen-Nürnberg, 91058 Erlangen, Germany; johannes.j.jaeger@fau.de (J.J.); matthias.lochmann@fau.de (M.L.); 2 Footballscience.net, 63303 Dreieich, Germany; 3 Sport Performance Research Institute New Zealand, AUT-University, Auckland 1142, New Zealand; 4 Centre for Research, Education, Innovation, and Intervention in Sport (CIFI2D), Faculty of Sport, University of Porto, 4150-564 Porto, Portugal; jm_silv@hotmail.com; 5 N2i, Instituto Politécnico da Maia, Universidade da Maia, 4475-690 Maia, Portugal; 6 Escola Superior Desporto e Lazer, Universidade Politécnica de Viana do Castelo, 4900-347 Viana do Castelo, Portugal

**Keywords:** field size, football, soccer, training, rehabilitation, return-to-play

## Abstract

**Background and Objectives**: The aim was to investigate the external load of different types of floaters and regular players in small-sided games (SSG) with different relative pitch sizes to inform the return-to-play process. **Methods**: Twenty-four professional soccer players from a Greek top-tier soccer club played SSG as regular players, internal floaters, and external floaters. Global-positioning units were utilized to investigate total distance (TD), as well as high-intensity running distances (HID), which was further divided into HID1 (15.0–19.9 km/h and HID2 (20.0–24.9 km/h). High-intensity running (HIR) was analyzed by distance and number of efforts (HIRnb) greater than 19.8 km/h. Velocity zones were categorized into zones such as 10.8–14.4 km/h (Zone3), 14.5–19.8 km/h (Zone4), 19.9–24.9 km/h (Zone5), >25 km/h (Zone6). Sprint metrics included number of sprints (Sprint/Nm) and sprint distance (Sprint/Dist). Acceleration bands were defined as high >4 m/s^2^ (ACC4), moderate 3–3.9 m/s^2^ (ACC3), and low 0–2.9 m/s^2^ (ACC2); and decelerations were defined as high <−4 m/s^2^ (DEC4), moderate −3.9–3 m/s^2^ (DEC3), and low −2.9–0 (DEC2 m/s^2^). All data were reported as relative value (standardized over a 1 min playing period). **Results**: Regular players showed the highest value, followed by internal floaters and external floaters for all variables, except for Zone5 distance. The majority of variables differed significantly between the regular players and the internal floaters except for TD, distance in Zone3 and Zone6, and number of ACC3, ACC4, and DEC4. ACC4 was the only variable found to be not significantly different between the regular players and the external floaters. In games with both floater types, internal floaters showed significantly higher values in all variables except for HIR, Zone6 distance, Sprint/Dist, Sprint/Nm, ACC3, ACC4, and DEC4. The comparison of external load variables between internal and external floaters when each player type was playing against regular players only revealed significantly higher values for all variables except for Sprint/Dist, Sprint/Nm, Zone6 distance, ACC3, and ACC4 for the internal floaters. Regression analyses were provided for all player types in the appropriated game format (RP with IF, RP with EF, RP with IF and EF). **Conclusions**: The on-field return-to-play process for individual players in the final stages incorporating SSG with different relative pitch sizes benefits from the utilization of different types of floaters. Increasing external load is ensured through starting with external floaters followed by internal floaters easing into the final role, the regular player role.

## 1. Introduction

Injury reduction remains a hot topic in soccer [1]. However, once an injury was experienced, the inherent return-to-play (RTP) process plays a crucial role in preventing re-injuries involving a gradual transition from rehabilitation to performance [2]. Based on the injury at hand, different RTP strategies and criteria for “common” [3,4] or more severe soccer injuries [5,6] are discussed throughout the scientific literature. While the initial rehabilitation process is tailored to the existing injury, the later stages of the rehabilitation process involve soccer-specific on-field training with various physical as well as technical and tactical aims [2]. This requires a progression from controlled drills to increasingly game-like situations, and the control–chaos continuum framework [7] provides a useful structure for this process. “Control” refers to predictable, coach-directed tasks, such as pre-planned changes of direction, or passing drills without opponent pressure [7]. “Chaos” refers to more unpredictable, game-like situations in which the player must react to teammates, opponents, and the ball, as in small-sided games (SSG) [7]. Within this framework, a constraints-led approach is used to adjust the difficulty and unpredictability of training tasks to finally participate in SSG with active opponents [7]. In this “high-chaos” stages, the player may partially or fully rejoin team training, where speed, decision-making, opponent pressure, and tactical demands resemble match play more closely [8]. This transition between stages can be facilitated by incorporating internal and/or external floaters [9], allowing the coach to ease the player back into the “chaotic” elements of team training and, importantly, allows the player to self-limit tempo within game-based activities [9]. As this “adapted” manner involves reduced overall load [10], load progression seemed to be key [11], and increments are determined through the player’s pre-injury GPS data [12].

Indeed, the limited scientific references with regards to floating players indicate that regular players achieved statistically greater total distance [13,14,15] and distance covered at 7–13.9, 14–17.9, and >19.8 km/h as well as a greater number of accelerations [13,14,15], decelerations [13,15], and sprints [13] compared to floaters. Furthermore, greater mechanical loads were likely to most-likely present in regular players compared to floaters [16], similarly to high-metabolic load [15]. Mechanical load was an overall measure of velocity changes and is calculated using >2 m/s^2^ accelerations, decelerations and change of direction events [16]. High-metabolic load represented the distance covered (m) by a player when their metabolic power (energy consumption per kilogram per second) was above the value of 25.5 W/kg [15]. Nevertheless, Asian-Clemente et al. [14] grouped the internal and external floaters together and therefore the scientific information seems incomplete. When comparing the two types of floaters with regular players as well as with each other, researchers showed that internal floaters experienced greater total [14] and relative [13] total distance and high-intensity distance at 10.8–19.99 km/h [13] and >15 km/h [13], as well as acceleration (2.0–4 m/s^2^) [13,14] and deceleration (−2.0–−4 m/s^2^) [13,14], compared to external floaters. This detail provided important implications for the aforementioned RTP process as players might be assigned different types of floater roles during different stages of the on-field rehabilitation. However, the given details are the sole scientific knowledge with regards to external load in different types of floaters during SSG. Furthermore, another task constraint, the relative pitch itself, was observed to influence the external load of the involved players [17,18,19] and therefore should also be taken into account whilst utilizing SSG during the RTP process. This is even more so because training load was consensually [2,4,20,21,22] seen as a key determinant in an effective on-field rehabilitation process [1]. Lacome and colleagues [16] were also able to provide some limited scientific knowledge in this area. More precisely, the greater pitch size provoked greater relative total distance (m/min) as well as relative high-speed (>14.4 km/h) running distance (m/min) in both floaters and regular players in game simulations and possession games. The changes for total distance were 20.2 and 11.9 m/min as well as 3.00 and 5.60 m/min for relative high-speed distance, respectively. The authors categorized small and large pitch sizes utilizing between 12 and 20 players on average for the game simulations and between 13 and 18 players on average for the possession games. Consequently, the relative pitch size was 65 m^2^ and 124 m^2^ for the game simulations and 61 m^2^ and 118 m^2^ for the possession games. With this limited scientific knowledge in this area, there seems to be a need for further investigations with regards to different types of floaters as well as utilizing different player numbers and the resulting relative pitch size to explore the external load of internal and external floaters with regards to regular players in adult soccer in different SSG. Therefore, the purpose was to investigate the external load of different types of floaters and regular players in SSG with different relative pitch sizes to inform the return-to-play process.

## 2. Materials and Methods

### 2.1. Participants

Twenty-four professional soccer players (24.9 ± 4.49 years, 181 ± 5.58 cm, 74.6 ± 5.70 kg) from the Greek 1st division “Super League” volunteered to participate in this study. All players trained at least once per day and were injury-free for the course of the data collection. The average training hours per week for the course of the data collection were 8.50 ± 1.48. Data arose from daily monitoring throughout the season and were obtained retrospectively from the club’s database. Furthermore, player data were collected as part of contractual obligations throughout the season [23] and, therefore, ethics committee clearance was not required [24]. However, all data were anonymized to ensure confidentiality and to prevent any identification of individual players or the club [25,26]. Nevertheless, the study was conducted strictly according to the Declaration of Helsinki. All players who took part in the matches and trained at least 80% of the training volume were selected for further analysis. Goalkeepers were not included in the study due to their specific demands. Despite the retrospective nature of the study, a sample size estimation was conducted using G*Power software (version 3.1). With a minimum effect size of 0.8, power of 0.8, and a *p*-value of 0.05, between 8–12 participants per training group were determined, depending on the variable.

### 2.2. Equipment

Global-positioning units (GPS) with a frequency of 10-Hz (Intense V 3.1.2., Insiders, Lausanne, Switzerland) were utilized to monitor the external load of the players for all training sessions. All devices were activated 30 min before data collection to allow acquisition of satellite signals as per manufacturer’s instructions. The GPS units were placed into a small pocket on the backside of a vest worn by the players accordingly to the manufacturer guidelines. After each training session, GPS data were downloaded using the respective software package (Intense V 3.1.2., Insiders, Lausanne, Switzerland). The units were deemed reliable and valid [27,28]; however, in-depth information is provided elsewhere. Four custom-made aluminum small mini goals with a size of 1.5 m (width) × 1 m (height) as well as FIFA-approved match balls (Puma, Herzogenaurach, Germany) with an official weight of 410–450 g and 68–70 cm circumference were used during the games. Several balls were placed around the pitch to restart the games as fast as possible when a ball went out-of-bounds.

### 2.3. Procedures

Generally, data were collected at the same time of day, and activity patterns in the 48 h prior to each data collection session were standardized around mode of training and daily structure. Participants received a 10–15 warm-up protocol on the pitch consisting of running, passing as well as static and dynamic flexibility exercises. The different SSG were coach-led and implemented after the warm-up. Specifications for the different games are stated in Table 1.

Whilst player number and pitch size differed from the individual games, other task constraints were kept constant. That is, four mini goals (1.5 m width × 1 m height) were used and a two-touch restriction was applied, except for the external floaters who were allowed only one-touch play. Internal floaters were not allowed to score. For all games the external floaters were distributed evenly outside the pitch in playing direction, while the internal floaters were allowed to roam freely inside the pitch restrictions.

### 2.4. Data Analysis

External load variables included total distance (TD) and high-intensity running distances (HID), which was further divided into HID1 (15.0–19.9 km/h and HID2 (20.0–24.9 km/h). High-intensity running (HIR) was analyzed by distance and number of efforts (HIRnb) greater than 19.8 km/h. Velocity zones were categorized as zones such as 10.8–14.4 km/h (Zone3), 14.5–19.8 km/h (Zone4), 19.9–24.9 km/h (Zone5), >25 km/h (Zone6). Sprint metrics included number of sprints (SprintNm) and sprint distance (Sprint/Dist). Acceleration bands were defined as high > 4 m/s^2^ (ACC4), moderate 3–3.9 m/s^2^ (ACC3), and low 0–2.9 m/s^2^ (ACC2). Similarly, deceleration bands were defined as high < −4 m/s^2^ (DEC4), moderate −3.9–−3 m/s^2^ (DEC3), and low −2.9–0 (DEC2 m/s^2^). All data were reported as relative value (standardized over a 1 min playing period); consequently, the different playing durations were accounted for over different formats and were statistically analyzed.

### 2.5. Statistical Analysis

All statistical analyses were performed in R (R Core Team, R Foundation for Statistical Computing, Vienna, Austria) using RStudio (Version 2026.4.6.1; Posit PBC, Boston, MA, USA). Descriptive data are presented as means with 95% confidence intervals (CIs). Differences in external load between player roles were examined separately for internal-only, external-only, and internal + external floater games using mixed-effects models, with player role as a fixed effect and player as a random intercept. Depending on outcome distribution, either linear mixed-effects models (LMMs) or generalized linear mixed-effects models (GLMMs) with a Tweedie distribution and log link were fitted. Model fit was evaluated using the Akaike information criterion (AIC), residual diagnostics, convergence, and dispersion. For each outcome, omnibus player-role effects were tested, followed by model-adjusted estimated marginal means (EMMs) with 95% CIs and predefined pairwise comparisons. Gaussian-model effects are reported as adjusted mean differences in the original unit, whereas Tweedie-model effects are presented as back-transformed ratios of expected means with corresponding percentage differences.

To directly compare floater positions across all available observations, additional LMMs were fitted with floater position (inside vs. outside) as a fixed effect and player as a random intercept. Model-estimated means with 95% CIs and adjusted mean differences were derived from the fixed-effect estimates, with inference based on Wald tests. The Benjamini–Hochberg false discovery rate (FDR) procedure was applied separately to all *p*-values of mixed-model tests.

Additionally, separate linear regression analyses were conducted to examine associations between relative pitch size (RPS) and each external-load variable within each game format and player role. Prior to interpretation, model assumptions were evaluated by visual inspection of scatterplots and residual-versus-fitted plots to assess linearity and homoscedasticity, and Q–Q plots to assess the distribution of residuals. Results are reported as unstandardized regression coefficients (B) with standard errors, *p* values, coefficients of determination (R^2^), and overall F statistics. Models were not estimated for outcomes with no variability. Statistical significance was set at *p* < 0.05 for all analyses [29].

## 3. Results

Table 2 shows the descriptive statistics for the different player type and variables across all small-sided games.

Regular players showed the highest value, followed by internal floaters and external floaters for all variables. Table 3 and Table 4 show the result of the differences in the mean between regular players and the internal and external floaters. The post hoc analysis is presented in Table 4 and the majority of variables differed significantly between the regular players and the internal floaters except for total distance, distance in Zone3 and Zone6, and number of accelerations (3–3.9 m/s^2^) and decelerations (−3–−3.9 m/s^2^). ACC4 was the only variable being not significantly different from the regular players in comparison to the external floaters.

The analysis between the two types of floaters revealed significance for all variables except for the following: HIR distance, Sprint/Dist, Sprint/Nm, Zone6 distance, number of ACC3 and ACC4 accelerations, and number of DEC4 decelerations (<−4 m/s^2^). The comparison of external load variables between internal and external floaters when each player type was playing against only regular players is presented in Table 5 and Table 6. Internal floaters showed significantly higher values for all variables except for Sprint/Dist, Sprint/Nm, Zone6 distance, and medium and high acceleration.

Table 7, Table 8 and Table 9 depicts the results of the regression analysis for all types of players.

Across all games, all variables were significantly affected by relative pitch size in regular players except for medium (−3.9–−3.0 m/s^2^) and high decelerations (>−4 m/s^2^). Furthermore, high acceleration (>4 m/s^2^) was not statistically significant for regular players involving internal floaters as well as both floater types. Lastly, distance in Zone6, Sprint/Dist, and low acceleration and deceleration and medium acceleration were significantly different for regular player in games with internal floaters, external floaters, and both types of floaters, respectively. Internal players’ external load showed significant changes with relative pitch size for the majority of variables (14 from 18). However, no significance was detected for HID, HID1, and Zone4 running for all games. Additionally, DEC4 and DEC3 were not statistically significant for games with regular players only, or for games involving regular players as well as external floaters. External players’ external load showed significant changes with relative pitch size in five and eight variables in games with regular players and internal floaters, and in games with regular players, respectively. Under both circumstances, HID, HID1, Zone3, and Zone4 were statistically significant variables. In addition, HID2, HIR, HIR (nb/min), and Zone5 were significantly different in games with regular players; and ACC3 was statistically significant in games with internal floaters and regular players. As no sprint metrics (Sprint/Dist and Sprint/Nm, Zone6) were acquired during the games for both floater types, the regression analysis was not performed for these variables. Table 7, Table 8 and Table 9 indicate the actual regression equation for significant variables for all types of players in the different game scenarios (regular players with internal floaters, regular players with external floaters, and regular players with both floater types). The letter “x” represents the desired relative pitch size that needs to be addressed to calculate the predicted external load (letter “y”) in the desired variable.

## 4. Discussion

The primary purpose was to investigate the external load of regular players, internal floaters, and external floaters during different small-sided games with various relative pitch sizes and their utility regarding the RTP-process. Generally, the physical load varied between the different players, and the load increased from external to internal floaters to regular players. However, variables at the top end of the velocity (Sprint and Zone6) and accelerometer categories were not significantly different between regular players and internal and external floaters. Generally, the results in this investigation are in line with previous research. Regular players reported statistically greater total greater total distance [13,14,15] and distance covered at 7–13.9, 14–17.9, and >19.8 km/h, as well as a greater number of sprints [13], accelerations [13,14,15], and decelerations [13,15]. Caution should be taken with regards to the latter one, as different and more defined acceleration thresholds were used in this study (ACC2 = 0–2 m/s^2^, ACC3 = 2–3 m/s^2^, and ACC4 = >4 m/s^2^) compared to Asian-Clemente et al. [14] (2.5–4 m/s^2^) and Casamichana et al. [15] (>3 m/s^2^). Nevertheless, the thresholds by Asian-Clemente et al. [14] were covered entirely, while the ones by Casamichana et al. [15] (>3 m/s^2^) were partly captured by one significant threshold (ACC3 from 3–4 m/s^2^) and a non-significant one (ACC4) in this investigation. It also seems reasonable to conclude that the greater mechanical load by the regular players reported by Lacome and colleagues [16] might also be present in this investigation, whilst not being quantified per se. However, mechanical load was defined as an overall measure calculated using >2 m/s^2^ accelerations, decelerations, and changes of direction events [16]. The respective accelerometer and deceleration variables in this investigation were significantly different between the regular players and the floaters as well.

In games involving all floater types, the analysis between the internal and external floaters revealed significance for all variables except for the following: HIR distance, Sprint/Dist, Sprint/Nm, number of medium (3–4 m/s^2^) and high accelerations (>4 m/s^2^), and number of high decelerations (<−4 m/s^2^). While the total distance was also significantly higher in internal floaters (compared to external ones) in two investigations [13,14], the acceleration and deceleration data in this study cannot necessarily confirm the information provided by the one of Asian-Clemente et al. [14]. As previously mentioned, the acceleration and decelerating thresholds were defined slightly differently; nevertheless internal floaters only showed non-significant differences compared to the external floaters. Rumpf et al. [13], however, had identical acceleration/deceleration thresholds and showed that internal floaters demonstrated significantly greater values than external floaters in relative values of TD, high-intensity distance (15.0–19.9 km/h; >15 km/h), distance running (10.8–19.8 km/h), low-intensity acceleration (0–2.9 m/s^2^), and low-intensity deceleration (−2.9–0 m/s^2^), as well as moderate deceleration (3.0–3.9 m/s^2^). The effect sizes ranged from trivial to very large. This is in accordance with this investigation; in addition, the numbers of HIR/min and Zone 5/min were also significantly different between the internal and external floaters. A secondary analysis regarding the two different floater types was performed comparing the external load variables between internal and external floaters when each player type was playing against regular players. Internal floaters showed significantly higher values for all variables except for Sprint/Dist, Sprint/NM, Zone6 distance, and medium and high acceleration, further confirming the values in accordance to the scientific literature and the differences between the two types of floaters.

Differences with this investigation might have been derived from a possible combination of multiple task constraints, such as touch restriction and possession games as well as different pitch sizes. Whilst this investigation utilized touch restrictions for the internal (two-touch) as well as the external (one-touch) floaters, Asian-Clemente et al. [14] utilized free play. In general, touch restrictions were reported to increase variables that are associated with greater external load [30], such as total distance [31], high-intensity running [31], and sprinting [31], but also time walking [31,32]. On the other hand, possession play was reported to increase the physical strain for players during SSG [33], and Asian-Clemente et al. [14] showed an increase in total and relative distance (m/min), total distance at speeds 0.1–12.9 km/h, and number of low (1–1.9 m/s^2^) and high (>2.5 m/s^2^) accelerations in comparison to mini-goals [34], as utilized in this investigation. Lastly, there were multiple pitch sizes and player numbers utilized in this investigation whilst Asian-Clemente et al. [14] only used one SSG setup, which most likely influenced outcome variables as well. Therefore, it seems challenging to disentangle the different results with regards to the accelerometer data from the existing literature in this manuscript, especially given the limited scientific evidence as well as the high variability of events (Coefficient Variation CV = 2.5–59%) related to changes in speed (acceleration/deceleration) in SSG [35]. Lastly, the external floaters in the study by Asian-Clemente [14] utilized 5 m of depth, while there was a smaller space (1 m) availability in this investigation. Both distances seemed arbitrary; however, 1 m felt sufficient to provide enough moving/playing space without compromising technical abilities of the players in this investigation.

Generally, the SSG in this investigation provided the regular players only with external load in sprinting (>25 km/h). This could be possibly explained by different task constraints between SSG compared to match play and its implication on number of sprints and sprinting speed [36]. It was stated that SSG conducted with match play-like terms of rules coincide with higher maximal sprinting speed [36]. Consequently, it seems reasonable to conclude that the SSG in this study inherited too many discrepancies with regards to match play and therefore failed to present an effect on sprint variables such as Zone6. Nevertheless, external load in high-velocity zones seemed of special interest with regards to the aforementioned RTP process and on-field rehabilitation, due to the fact that high weekly sprinting load [37] as well as large and rapid increases in sprinting distance was observed to increase odds of injury [38]. Consequently, staff involved with the RTP process need to take into account that both floater types are subject to little exposure regarding sprinting distance (>25 km/h), at least based on the result in this investigation and inherited task constraints. However, the lower the running velocity, the closer the external load compared to regular players. More precisely, decreasing velocity, from Zone5 (19.9–24.9 km/h) to Zone4 (14.5–19.8 km/h) and Zone3 (10.8–14.4 km/h), resulted in less difference in external load with regards to the regular players. Therefore, the different types of floaters and their roles might serve as a distinct period in an individual’s player RTP process. More precisely and despite the fact that internal floaters seemed to be more “game specific” than external floaters [16], external floaters might be the preferred choice in earlier stages of the on-field RTP process. This role inherits less chaos and therefore greater predictability and planning. Accordingly, the regression equations in this study provide a quantitative description of the observed relationships between floater role, relative pitch size, and different external load variables and may help practitioners contextualize expected demands during RTP progression. For example, this investigation provides information that explains starting in reverse from the external load (total m/min) towards the desired relative pitch size with the provided regression equation, if the entire SSG needs to be tailored to the single individual. However, only the variables that are significantly different in the regression analysis are described and can therefore be used to plan training load for different roles during SSG.

This scientific investigation is not without limitations. Firstly and unfortunately, based on the data in this investigation, it seems impossible to plan external sprinting load in the RTP process, as the internal and external floaters did not experience any of the aforementioned load during the games. Secondly, the players used as internal and external floaters in this investigation were not injured. Consequently, truly injured players in the RTP process might experience the SSG dynamics psycho-physiologically differently, which might affect movement and therefore external load. The variables investigated included velocities above 10.8 km/h and therefore might miss important external load (<10.8 km/h) distances that might not be physiologically taxing for individual players, but might, however, add to the overall training load, especially for the external floaters. The regression equations were derived and evaluated within the present sample and were not subjected to internal validation. Their predictive accuracy and generalizability therefore remain unknown, and prospective validation in independent samples is required before they can be used for RTP decision-making. Additionally, coaches and staff involved in the on-field RTP process should be cognizant of the task constraints in this investigation. Deviating from touch restriction (one-touch for external and two-touch for internal floaters) and four-goal games without goalkeepers might alter the game dynamics and consequently the external load of rehabilitating players.

## 5. Conclusions

The on-field RTP process of individual players in the final stages incorporating SSG with different relative pitch sizes can benefit from the utilization of different types of floaters. Increasing external load is ensured through starting with external floaters followed by internal floaters easing into the final role, the regular player role. However, individual pre-injury external load metrics should be taken into account whilst planning the individual RTP process. The regression analysis for each role and the different variables in this investigation enable staff to develop better programming.

## Figures and Tables

**Table 1:** Specification of different SSG.

Player Format (Number of Observations)	Pitch Size (Length × Width)	Relative Pitch Size (m^2^)	Game Duration (min)
3 vs. 3 + 3	20 × 18	60	2 min
Regular players (18)			
External floater (11)			
4 vs. 4 + 1	40 × 40	200	2.45 min
Regular players (38)			
Internal floater (3)			
4 vs. 4 + 2	40 × 40	160	3.5 min
Regular players (11)			
Internal floater (1)			
4 vs. 4 + 4	20 × 18	45	2 min
Regular players (41)			
External floater (16)			
4 vs. 4 + 4	20 × 18	45	2.5 min
Regular players (39)			
External floater (20)			
4 vs. 4 + 4 + 2	40 × 30	120	4 min
Regular players (60)			
External floater (20)			
Internal floater (7)			
5 vs. 5 + 1	40 × 40	145	3 min
Regular players (12)			
Internal floater (1)			
5 vs. 5 + 4	40 × 30	120	2 min
Regular players (60)			
External floater (19)			
6 vs. 6 + 1	40 × 30	92	4 min
Regular players (39)			
Internal floater (2)			
6 vs. 6 + 1	60 × 40	184	4 min
Regular players (38)			
Internal floater (3)			
6 vs. 6 + 12	40 × 30	100	2 min
Regular players (122)			
External floater (102)			
6 vs. 6 + 6 +1	40 × 18	55	2 min
Regular players (107)			
External floater (51)			
Internal floater (11)			
6 vs. 6 + 6	45 × 40	150	3 min
Regular players (38)			
External floater (14)			
6 vs. 6 + 6	45 × 40	150	4 min
Regular players (39)			
External floater (18)			
7 vs. 7 + 1	60 × 40	171	4 min
Regular players (14)			
Internal floater (1)			
7 vs. 7 + 1	60 × 40	160	3 min
Regular players (41)			
Internal floater (2)			
7 vs. 7 + 1	60 × 40	160	4.5 min
Regular players (26)			
Internal floater (2)			
7 vs. 7 + 1	60 × 40	160	6 min
Regular players (28)			
Internal floater (3)			
7 vs. 7 + 7 + 1	50 × 40	133	3 min
Regular players (39)			
External floater (18) Internal floater (3)			
8 vs. 8 + 8	40 × 40	100	2.5 min
Regular players (46)			
External floater (22)			
8 vs. 8 + 8	50 × 40	125	2.50 min
Regular players (46)			
External floater (23)			
8 vs. 8 + 8	50 × 40	125	3.50 min
Regular players (44)			
External floater (22)			

**Table 2:** Descriptive statistics for all variables and groups.

Variable	Player Type	Number of Observations	Mean ± Std	95% Lower Confidence Interval	95% Upper Confidence Interval	Min	Max
TD (m/min)	Internal floaters	39	112 ± 39.4	99.3	125	19.4	184
	External floaters	358	27.9 ± 17.5	26.1	29.7	0.00	88.5
	Regular players	945	124 ± 34.4	122	126	11.1	200
HID (m/min)	Internal floaters	39	15.4 ± 20.2	8.85	21.9	0.00	90.5
	External floaters	358	0.32 ± 1.00	0,22	0.43	0.00	7.06
	Regular players	945	26.3 ± 19.5	25.1	27.6	0.00	133
HID1 (m/min)	Internal floaters	39	13.3 ± 16.5	7.93	18.6	0.00	66.9
	External floaters	358	0.30 ± 0.89	0.21	0.39	0.00	6.59
	Regular players	945	20.7 ± 15.3	19.8	21.7	0.00	77.1
HID2 (m/min)	Internal floaters	39	2.11 ± 5.10	0.46	3.76	0.00	24.0
	External floaters	358	0.03 ± 0.22	0.00	0.05	0.00	2.50
	Regular players	945	4.86 ± 5.69	4.50	5.22	0.00	35.2
HIR (m/min)	Internal floaters	39	2.32 ± 5.43	0.56	4.08	0.00	25.1
	External floaters	358	0.03 ± 0.25	0.01	0.06	0.00	2.86
	Regular players	945	6.03 ± 7.97	5.52	6.53	0.00	125
HIR (nb/min)	Internal floaters	39	0.21 ± 0.40	0.08	0.34	0.00	1.45
	External floaters	358	0.01 ± 0.04	0.00	0.01	0.00	0.41
	Regular players	945	0.54 ± 0.55	0.50	0.57	0.00	3.75
Sprint/Dist (m/min)	Internal floaters	39	0.00 ± 0.00	0.00	0.00	0.00	0.00
	External floaters	358	0.00 ± 0.00	0.00	0.00	0.00	0.00
	Regular players	945	0.73 ± 3.69	0.50	0.97	0.00	90.1
Sprint/Nm (Nm/min)	Internal floaters	39	0.00 ± 0.00	0.00	0.00	0.00	0.00
	External floaters	358	0.00 ± 0.00	0.00	0.00	0.00	0.00
	Regular players	945	0.06 ± 0.15	0.05	0.06	0.00	1.00
ACC2 (nb/min)	Internal floaters	39	3.78 ± 2.37	3.01	4.55	0.00	10.9
	External floaters	358	1.07 ± 1.02	0.96	1.17	0.00	5.95
	Regular players	945	5.24 ± 2.41	5.09	5.40	0.00	15.9
ACC3 (nb/min)	Internal floaters	39	0.35 ± 0.47	0.20	0.50	0.00	1.85
	External floaters	358	0.35 ± 0.48	0.30	0.40	0.00	3.12
	Regular players	945	0.75 ± 0.78	0.70	0.80	0.00	5.16
ACC4 (nb/min)	Internal floaters	39	0.06 ± 0.18	0.00	0.12	0.00	0.88
	External floaters	358	0.10 ± 0.21	0.07	0.12	0.00	1.35
	Regular players	945	0.07 ± 0.19	0.06	0.08	0.00	1.86
DEC2 (nb/min)	Internal floaters	39	3.08 ± 1.32	2.65	3.51	0.29	5.25
	External floaters	358	0.43 ± 0.77	0.35	0.51	0.00	6.19
	Regular players	945	4.44 ± 1.80	4.32	4.55	0.00	11.5
DEC3 (nb/min)	Internal floaters	39	0.54 ± 0.41	0.40	0.67	0.00	1.45
	External floaters	358	0.07 ± 0.22	0.05	0.10	0.00	1.70
	Regular players	945	1.08 ± 0.79	1.03	1.13	0.00	4.76
DEC4 (nb/min)	Internal floaters	39	0.08 ± 0.18	0.02	0.13	0.00	0.73
	External floaters	358	0.01 ± 0.08	0.01	0.02	0.00	0.85
	Regular players	945	0.23 ± 0.34	0.21	0.25	0.00	2.66
Zone3 (m/min)	Internal floaters	39	25.3 ± 20.7	18.6	32.0	0.00	70.7
	External floaters	358	1.04 ± 2.45	0.78	1.29	0.00	20.8
	Regular players	945	29.1 ± 14.3	28.2	30.1	0.00	73.7
Zone4 (m/min)	Internal floaters	39	16.2 ± 19.3	9.89	22.4	0.00	72.4
	External floaters	358	0.38 ± 1.06	0.27	0.49	0.00	7.98
	Regular players	945	24.2 ± 17.1	23.1	25.3	0.00	89.3
Zone5 (m/min)	Internal floaters	39	2.32 ± 5.43	0.56	4.08	0.00	25.1
	External floaters	358	0.03 ± 0.25	0.01	0.06	0.00	2.86
	Regular players	945	5.35 ± 6.09	4.96	5.74	0.00	35.7
Zone6 (m/min)	Internal floaters	39	0.00 ± 0.00	0.00	0.00	0.00	0.00
	External floaters	357	0.00 ± 0.00	0.00	0.00	0.00	0.00
	Regular players	945	0.02 ± 0.11	0.01	0.03	0.00	2.30

**Table 3:** LMM main effects of player role and model-adjusted estimated marginal means.

Outcome Variable	Regular Player	Internal Floater	External Floater	Omnibus Player-Role Test	*p*
Games with regular players and internal floaters					
TD (m/min)	124 (81.1–167)	128 (84.9–171)	—	F(1) = 0.71	0.424
HID (m/min)	35.3 (31.7–39.5)	21.0 (15.9–27.6)	—	χ^2^(1) = 15.37	**0.002**
HID1 (m/min)	27.2 (24.1–30.6)	18.7 (13.78–25.3)	—	χ^2^(1) = 6.76	**0.025**
HID2 (m/min)	6.45 (5.14–8.08)	3.12 (1.82–5.35)	—	χ^2^(1) = 7.32	**0.023**
HIR distance (m/min)	8.17 (6.15–10.9)	2.79 (1.50–5.19)	—	χ^2^(1) = 12.27	**0.005**
HIR (nb/min)	0.70 (0.63–0.77)	0.33 (0.21–0.54)	—	χ^2^(1) = 8.49	**0.023**
Sprint/Dist (m/min)	0.70 (0.36–1.13)	0.00 (0.00–0.57)	—	F(1) = 4.76	**0.044**
Sprint/Nm (nb/min)	0.08 (0.05–0.12)	0.00 (0.00–0.06)	—	F(1) = 6.89	**0.025**
ACC2 (nb/min)	4.44 (3.62–5.26)	3.17 (1.88–4.47)	—	F(1) = 6.47	**0.027**
ACC3 (nb/min)	0.49 (0.41–0.58)	0.38 (0.22–0.65)	—	χ^2^(1) = 1.30	0.298
ACC4 (nb/min)	0.03 (0.01–0.07)	0.06 (0.02–0.19)	—	χ^2^(1) = 1.36	0.298
DEC2 (nb/min)	3.95 (3.36–4.53)	3.13 (2.25–4.00)	—	F(1) = 5.64	**0.035**
DEC3 (nb/min)	0.90 (0.73–1.10)	0.61 (0.42–0.90)	—	χ^2^(1) = 5.03	**0.042**
DEC4 (nb/min)	0.20 (0.17–0.25)	0.06 (0.02–0.18)	—	χ^2^(1) = 4.25	0.052
Zone3 distance (m/min)	33.8 (28.4–40.3)	33.0 (25.3–42.9)	—	χ^2^(1) = 0.09	0.760
Zone4 distance (m/min)	31.4 (27.8–35.3)	22.9 (17.2–30.67)	—	χ^2^(1) = 5.52	**0.035**
Zone5 distance (m/min)	7.11 (5.72–8.84)	3.50 (2.09–5.84)	—	χ^2^(1) = 7.61	**0.023**
Zone6 distance (m/min)	0.06 (0.01–0.11)	0.00 (0.00–0.11)	—	F(1) = 1.16	0.315
Games with regular players and external floaters					
TD (m/min)	124 (115–133)	—	29.7 (20.8–38.6)	F(1) = 2692.85	**<0.001**
HID (m/min)	17.2 (11.7–25.4)	—	0.11 (0.06–0.21)	χ^2^(1) = 369.15	**<0.001**
HID1 (m/min)	14.2 (9.73–20.8)	—	0.11 (0.06–0.20)	χ^2^(1) = 348.90	**<0.001**
HID2 (m/min)	1.65 (0.74–3.71)	—	0.00 (0.00–0.01)	χ^2^(1) = 47.88	**<0.001**
HIR distance (m/min)	2.06 (0.95–4.44)	—	0.00 (0.00–0.02)	χ^2^(1) = 53.40	**<0.001**
HIR (nb/min)	0.22 (0.11–0.45)	—	0.00 (0.00–0.01)	χ^2^(1) = 60.51	**<0.001**
Sprint/Dist (m/min)	0.30 (0.21–0.39)	—	0.00 (0.00–0.08)	F(1) = 39.37	**<0.001**
Sprint/Nm (nb/min)	0.05 (0.03–0.06)	—	0.00 (0.00–0.01)	F(1) = 47.11	**<0.001**
ACC2 (nb/min)	5.17 (4.78–5.56)	—	1.19 (0.79–1.58)	F(1) = 651.80	**<0.001**
ACC3 (nb/min)	0.71 (0.57–0.88)	—	0.38 (0.30–0.48)	χ^2^(1) = 42.73	**<0.001**
ACC4 (nb/min)	0.05 (0.03–0.08)	—	0.07 (0.05–0.11)	χ^2^(1) = 2.95	0.086
DEC2 (nb/min)	4.47 (4.18–4.77)	—	0.47 (0.17–0.77)	F(1) = 1125.13	**<0.001**
DEC3 (nb/min)	1.04 (0.88–1.24)	—	0.07 (0.05–0.10)	χ^2^(1) = 263.47	**<0.001**
DEC4 (nb/min)	0.20 (0.16–0.27)	—	0.01 (0.01–0.02)	χ^2^(1) = 80.72	**<0.001**
Zone3 distance (m/min)	25.6 (21.3–30.9)	—	0.82 (0.61–1.09)	χ^2^(1) = 849.77	**<0.001**
Zone4 distance (m/min)	17.1 (12.0–24.4)	—	0.14 (0.08–0.26)	χ^2^(1) = 374.55	**<0.001**
Zone5 distance (m/min)	1.85 (0.83–4.10)	—	0.00 (0.00–0.02)	χ^2^(1) = 53.94	**<0.001**
Zone6 distance (m/min)	0.01 (0.01–0.02)	—	0.00 (0.00–0.01)	F(1) = 9.56	**0.002**
Games with regular players, internal floaters, and external floaters					
TD (m/min)	121 (99.8–142)	100 (78.0–122)	36.2 (15.1–57.3)	F(2) = 520.79	**<0.001**
HID (m/min)	20.4 (15.7–26.6)	6.43 (3.23–12.8)	0.60 (0.37–0.97)	χ^2^(2) = 110.60	**<0.001**
HID1 (m/min)	16.8 (12.7–22.2)	5.82 (2.88–11.8)	0.56 (0.34–0.92)	χ^2^(2) = 105.43	**<0.001**
HID2 (m/min)	3.22 (2.84–3.64)	0.51 (0.07–1.13)	0.03 (0.00–0.13)	F(2) = 109.69	**<0.001**
HIR distance (m/min)	3.73 (3.29–4.21)	0.57 (0.11–1.21)	0.03 (0.00–0.13)	F(2) = 126.30	**<0.001**
HIR (nb/min)	0.50 (0.43–0.58)	0.12 (0.00–0.26)	0.00 (0.00–0.06)	F(2) = 91.53	**<0.001**
Sprint/Dist (m/min)	0.16 (0.04–0.30)	0.00 (0.00–0.17)	0.00 (0.00–0.11)	F(2) = 6.96	**0.001**
Sprint/Nm (nb/min)	0.03 (0.01–0.05)	0.00 (0.00–0.04)	0.00 (0.00–0.02)	F(2) = 6.07	**0.003**
ACC2 (nb/min)	5.72 (4.97–6.46)	4.53 (2.73–6.34)	1.59 (0.84–2.34)	F(2) = 171.55	**<0.001**
ACC3 (nb/min)	0.80 (0.59–1.09)	0.32 (0.10–1.08)	0.51 (0.36–0.71)	χ^2^(2) = 34.51	**<0.001**
ACC4 (nb/min)	4.58 (3.87–5.28)	3.31 (2.13–4.50)	0.70 (−0.01–1.40)	F(2) = 337.25	**<0.001**
DEC2 (nb/min)	1.13 (0.83–1.53)	0.55 (0.21–1.45)	0.16 (0.10–0.27)	χ^2^(2) = 122.05	**<0.001**
DEC3 (nb/min)	27.2 (19.4–38.3)	16.0 (8.72–29.2)	1.61 (1.02–2.54)	χ^2^(2) = 306.95	**<0.001**
DEC4 (nb/min)	20.1 (15.4–26.2)	7.14 (3.72–13.7)	0.72 (0.46–1.14)	χ^2^(2) = 141.62	**<0.001**
Zone3 distance (m/min)	3.59 (3.18–4.05)	0.57 (0.11–1.20)	0.03 (0.00–0.13)	F(2) = 126.34	**<0.001**
Zone4 distance (m/min)	0.01 (0.00–0.01)	0.00 (0.00–0.01)	0.00 (0.00–0.01)	F(2) = 5.37	**0.006**
Zone5 distance (m/min)	0.08 (0.04–0.15)	0.06 (0.01–0.49)	0.17 (0.10–0.29)	χ^2^(2) = 2.89	0.236
Zone6 distance (m/min)	0.22 (0.15–0.34)	0.03 (0.00–1.33)	0.02 (0.01–0.06)	χ^2^(2) = 38.24	**<0.001**

**Table 4:** LMM post hoc analysis.

Outcome Variable	Contrast	Effect Estimate (95% CI)	*p*
Games with regular players and internal floaters			
TD (m/min)	Internal floater vs. regular player	MD = 3.95 m·min^−1^ (95% CI −4.93–12.82)	0.400
HID (m/min)	Internal floater vs. regular player	Ratio = 0.593 (95% CI 0.456–0.770), −40.7%	**0.002**
HID1 (m/min)	Internal floater vs. regular player	Ratio = 0.688 (95% CI 0.513–0.921), −31.2%	**0.035**
HID2 (m/min)	Internal floater vs. regular player	Ratio = 0.485 (95% CI 0.292–0.804), −51.5%	**0.020**
HIR distance (m/min)	Internal floater vs. regular player	Ratio = 0.342 (95% CI 0.193–0.604), −65.8%	**0.002**
HIR (nb/min)	Internal floater vs. regular player	Ratio = 0.479 (95% CI 0.299–0.769), −52.1%	**0.015**
Sprint/Dist (m/min)	Internal floater vs. regular player	Ratio= 0.566 (95% CI 0.348–0.922), −43.4%	**0.048**
Sprint/Nm (nb/min)	Internal floater vs. regular player	Ratio= 0.913 (95% CI 0.854–0.975), −8.7%	**0.025**
ACC2 (nb/min)	Internal floater vs. regular player	MD = −1.27 n·min^−1^ (95% CI −2.41 to −0.13)	**0.048**
ACC3 (nb/min)	Internal floater vs. regular player	Ratio = 0.780 (95% CI 0.455–1.338), −22.0%	0.400
ACC4 (nb/min)	Internal floater vs. regular player	MD = −0.82 n·min^−1^ (95% CI −1.57 to −0.06)	**0.048**
DEC2 (nb/min)	Internal floater vs. regular player	Ratio = 0.684 (95% CI 0.483–0.968), −31.6%	**0.048**
DEC3 (nb/min)	Internal floater vs. regular player	Ratio = 0.974 (95% CI 0.787–1.204), −2.6%	0.805
DEC4 (nb/min)	Internal floater vs. regular player	Ratio = 0.732 (95% CI 0.555–0.966), −26.8%	**0.048**
Zone3 distance (m/min)	Internal floater vs. regular player	Ratio = 0.491 (95% CI 0.304–0.795), −50.9%	**0.019**
Zone4 distance (m/min)	Internal floater vs. regular player	Ratio= 0.943 (95% CI 0.849–1.048), −5.7%	0.323
Zone5 distance (m/min)	Internal floater vs. regular player	Ratio = 1.958 (95% CI 0.777–4.937), +95.8%	0.193
Zone6 distance (m/min)	Internal floater vs. regular player	Ratio = 0.280 (95% CI 0.086–0.907), −72.0%	**0.048**
Games with regular players and external floaters			
TD (m/min)	External floater vs. regular player	MD = −94.11 m·min^−1^ (95% CI −97.66 to −90.57)	**<0.001**
HID (m/min)	External floater vs. regular player	Ratio = 0.006 (95% CI 0.004–0.011), −99.4%	**<0.001**
HID1 (m/min)	External floater vs. regular player	Ratio = 0.008 (95% CI 0.005–0.013), −99.2%	**<0.001**
HID2 (m/min)	External floater vs. regular player	Ratio = 0.001 (95% CI 0.000–0.005), −99.9%	**<0.001**
HIR distance (m/min)	External floater vs. regular player	Ratio = 0.001 (95% CI 0.000–0.008), −99.9%	**<0.001**
HIR (nb/min)	External floater vs. regular player	Ratio = 0.007 (95% CI 0.002–0.024), −99.3%	**<0.001**
Sprint/Dist (m/min)	External floater vs. regular player	Ratio= 0.770 (95% CI 0.711–0.834), −23.0%	**<0.001**
Sprint/Nm (nb/min)	External floater vs. regular player	Ratio= 0.955 (95% CI 0.943–0.967), −4.5%	**<0.001**
ACC2 (nb/min)	External floater vs. regular player	MD = −3.98 n·min^−1^ (95% CI −4.29 to −3.68)	**<0.001**
ACC3 (nb/min)	External floater vs. regular player	Ratio = 0.534 (95% CI 0.441–0.646), −46.6%	**<0.001**
ACC4 (nb/min)	External floater vs. regular player	MD = −4.01 n·min^−1^ (95% CI −4.24 to −3.77)	**<0.001**
DEC2 (nb/min)	External floater vs. regular player	Ratio = 0.065 (95% CI 0.047–0.090), −93.5%	**<0.001**
DEC3 (nb/min)	External floater vs. regular player	Ratio = 0.032 (95% CI 0.025–0.040), −96.8%	**<0.001**
DEC4 (nb/min)	External floater vs. regular player	Ratio = 0.008 (95% CI 0.005–0.013), −99.2%	**<0.001**
Zone3 distance (m/min)	External floater vs. regular player	Ratio = 0.001 (95% CI 0.000–0.008), −99.9%	**<0.001**
Zone4 distance (m/min)	External floater vs. regular player	Ratio= 0.988 (95% CI 0.980–0.995), −1.2%	**0.002**
Zone5 distance (m/min)	External floater vs. regular player	Ratio = 1.460 (95% CI 0.961–2.219), +46.0%	0.076
Zone6 distance (m/min)	External floater vs. regular player	Ratio = 0.061 (95% CI 0.033–0.111), −93.9%	**<0.001**
Games with regular players, internal floaters, and external floaters			
TD (m/min)	Internal floater vs. regular player	MD = −20.95 m·min^−1^ (95% CI −36.88 to −5.03)	**0.018**
	External floater vs. regular player	MD = −84.73 m·min^−1^ (95% CI −90.24 to −79.23)	**<0.001**
	External floater vs. internal floater	MD = −63.78 m·min^−1^ (95% CI −79.72 to −47.85)	**<0.001**
HID (m/min)	Internal floater vs. regular player	Ratio = 0.315 (95% CI 0.162–0.609), −68.5%	**0.001**
	External floater vs. regular player	Ratio = 0.029 (95% CI 0.019–0.045), −97.1%	**<0.001**
	External floater vs. internal floater	Ratio = 0.093 (95% CI 0.043–0.202), −90.7%	**<0.001**
HID1 (m/min)	Internal floater vs. regular player	Ratio = 0.346 (95% CI 0.177–0.676), −65.4%	**0.003**
	External floater vs. regular player	Ratio = 0.033 (95% CI 0.022–0.051), −96.7%	**<0.001**
	External floater vs. internal floater	Ratio = 0.096 (95% CI 0.044–0.211), −90.4%	**<0.001**
HID2 (m/min)	Internal floater vs. regular player	Ratio= 0.358 (95% CI 0.251–0.510), −64.2%	**<0.001**
	External floater vs. regular player	Ratio= 0.243 (95% CI 0.214–0.277), −75.7%	**<0.001**
	External floater vs. internal floater	Ratio= 0.679 (95% CI 0.476–0.968), −32.1%	**0.048**
HIR distance (m/min)	Internal floater vs. regular player	Ratio= 0.331 (95% CI 0.231–0.474), −66.9%	**<0.001**
	External floater vs. regular player	Ratio= 0.217 (95% CI 0.191–0.248), −78.3%	**<0.001**
	External floater vs. internal floater	Ratio= 0.656 (95% CI 0.458–0.941), −34.4%	**0.034**
HIR (nb/min)	Internal floater vs. regular player	Ratio= 0.743 (95% CI 0.662–0.835), −25.7%	**<0.001**
	External floater vs. regular player	Ratio= 0.668 (95% CI 0.640–0.696), −33.2%	**<0.001**
	External floater vs. internal floater	Ratio= 0.898 (95% CI 0.800–1.009), −10.2%	0.089
Sprint/Dist (m/min)	Internal floater vs. regular player	Ratio= 0.843 (95% CI 0.717–0.992), −15.7%	0.056
	External floater vs. regular player	Ratio= 0.856 (95% CI 0.807–0.908), −14.4%	**<0.001**
	External floater vs. internal floater	Ratio= 1.016 (95% CI 0.864–1.194), +1.6%	0.880
Sprint/Nm (nb/min)	Internal floater vs. regular player	Ratio= 0.964 (95% CI 0.928–1.002), −3.6%	0.085
	External floater vs. regular player	Ratio= 0.967 (95% CI 0.953–0.980), −3.3%	**<0.001**
	External floater vs. internal floater	Ratio= 1.002 (95% CI 0.965–1.041), +0.2%	0.918
ACC2 (nb/min)	Internal floater vs. regular player	MD = −1.18 n·min^−1^ (95% CI −3.01 to 0.65)	0.241
	External floater vs. regular player	MD = −4.13 n·min^−1^ (95% CI −4.75 to −3.50)	**<0.001**
	External floater vs. internal floater	MD = −2.94 n·min^−1^ (95% CI −4.78 to −1.11)	**0.003**
ACC3 (nb/min)	Internal floater vs. regular player	Ratio = 0.401 (95% CI 0.118–1.362), −59.9%	0.174
	External floater vs. regular player	Ratio = 0.631 (95% CI 0.452–0.882), −36.9%	**0.012**
	External floater vs. internal floater	Ratio = 1.576 (95% CI 0.460–5.400), +57.6%	0.522
ACC4 (nb/min)	Internal floater vs. regular player	Ratio = 0.745 (95% CI 0.082–6.743), −25.5%	0.850
	External floater vs. regular player	Ratio = 2.204 (95% CI 1.147–4.235), +120.4%	**0.030**
	External floater vs. internal floater	Ratio = 2.958 (95% CI 0.337–25.970), +195.8%	0.371
DEC2 (nb/min)	Internal floater vs. regular player	MD = −1.26 n·min^−1^ (95% CI −2.40 to −0.13)	**0.045**
	External floater vs. regular player	MD = −3.88 n·min^−1^ (95% CI −4.28 to −3.48)	**<0.001**
	External floater vs. internal floater	MD = −2.62 n·min^−1^ (95% CI −3.75 to −1.48)	**<0.001**
DEC3 (nb/min)	Internal floater vs. regular player	Ratio = 0.489 (95% CI 0.187–1.279), −51.1%	0.174
	External floater vs. regular player	Ratio = 0.142 (95% CI 0.088–0.229), −85.8%	**<0.001**
	External floater vs. internal floater	Ratio = 0.291 (95% CI 0.103–0.826), −70.9%	**0.032**
DEC4 (nb/min)	Internal floater vs. regular player	Ratio = 0.126 (95% CI 0.003–6.057), −87.4%	0.340
	External floater vs. regular player	Ratio = 0.102 (95% CI 0.041–0.256), −89.8%	**<0.001**
	External floater vs. internal floater	Ratio = 0.812 (95% CI 0.016–42.304), −18.8%	0.918
Zone3 distance (m/min)	Internal floater vs. regular player	Ratio = 0.586 (95% CI 0.347–0.988), −41.4%	0.063
	External floater vs. regular player	Ratio = 0.059 (95% CI 0.042–0.083), −94.1%	**<0.001**
	External floater vs. internal floater	Ratio = 0.101 (95% CI 0.055–0.185), −89.9%	**<0.001**
Zone4 distance (m/min)	Internal floater vs. regular player	Ratio = 0.355 (95% CI 0.191–0.658), −64.5%	**0.002**
	External floater vs. regular player	Ratio = 0.036 (95% CI 0.024–0.053), −96.4%	**<0.001**
	External floater vs. internal floater	Ratio = 0.101 (95% CI 0.049–0.208), −89.9%	**<0.001**
Zone5 distance (m/min)	Internal floater vs. regular player	Ratio= 0.341 (95% CI 0.240–0.485), −65.9%	**<0.001**
	External floater vs. regular player	Ratio= 0.224 (95% CI 0.197–0.254), −77.6%	**<0.001**
	External floater vs. internal floater	Ratio= 0.656 (95% CI 0.461–0.934), −34.4%	**0.032**
Zone6 distance (m/min)	Internal floater vs. regular player	Ratio= 0.992 (95% CI 0.984–1.001), −0.8%	0.085
	External floater vs. regular player	Ratio= 0.993 (95% CI 0.990–0.996), −0.7%	**<0.001**
	External floater vs. internal floater	Ratio= 1.001 (95% CI 0.992–1.009), +0.1%	0.880

**Table 5:** Comparison of external-load outcomes between internal and external floaters.

Outcome	Internal Floaters, EMM (95% CI)	External Floaters, EMM (95% CI)	Mean Difference (95% CI)	SE	z	*p*
TD (m/min)	110.6 (103.6–117.6)	28.0 (24.8–31.1)	82.6 (75.5–89.7)	3.6	22.86	* <0.001
HID (m/min)	15.4 (13.3–17.5)	0.3 (−0.6–1.1)	15.1 (13.0–17.3)	1.1	13.81	* <0.001
HID1 (m/min)	13.3 (11.6–15.0)	0.3 (−0.4–1.0)	13.0 (11.3–14.8)	0.9	14.48	* <0.001
HID2 (m/min)	2.1 (1.6–2.6)	0.0 (−0.1–0.2)	2.1 (1.6–2.6)	0.3	7.74	* <0.001
HIR (m/min)	2.3 (1.8–2.9)	0.0 (−0.1–0.2)	2.3 (1.7–2.8)	0.3	7.96	* <0.001
HIR (nb/min)	0.21 (0.17–0.26)	0.01 (−0.01–0.02)	0.21 (0.16–0.25)	0.02	9.10	* <0.001
Sprint/Dist (m/min)	0.0 (0.0–0.0)	0.0 (0.0–0.0)	0.0 (0.0–0.0)	0.0	0.00	1.000
Sprint/Nm (nb/min)	0.00 (0.00–0.00)	0.00 (0.00–0.00)	0.00 (0.00–0.00)	0.00	0.00	1.000
Zone3 (m/min)	24.7 (22.4–27.0)	1.1 (0.1–2.1)	23.6 (21.3–26.0)	1.2	19.77	* <0.001
Zone4 (m/min)	16.1 (14.1–18.2)	0.3 (−0.5–1.2)	15.8 (13.7–17.9)	1.1	14.98	* <0.001
Zone5 (m/min)	2.3 (1.8–2.9)	0.0 (−0.1–0.2)	2.3 (1.7–2.8)	0.3	7.96	* <0.001
Zone6 (m/min)	0.0 (0.0–0.0)	0.0 (0.0–0.0)	0.0 (0.0–0.0)	0.0	0.00	1.000
ACC2 (nb/min)	3.86 (3.45–4.27)	1.05 (0.88–1.22)	2.81 (2.39–3.22)	0.21	13.25	* <0.001
ACC3 (nb/min)	0.37 (0.21–0.53)	0.35 (0.29–0.41)	0.02 (−0.14–0.18)	0.08	0.27	0.942
ACC4 (nb/min)	0.06 (−0.00–0.13)	0.10 (0.07–0.12)	−0.03 (−0.10–0.04)	0.03	−0.89	0.479
DEC2 (nb/min)	3.08 (2.82–3.35)	0.43 (0.34–0.52)	2.66 (2.38–2.93)	0.14	18.79	* <0.001
DEC3 (nb/min)	0.54 (0.46–0.62)	0.07 (0.05–0.10)	0.46 (0.38–0.55)	0.04	11.18	* <0.001
DEC4 (nb/min)	0.08 (0.05–0.10)	0.01 (0.00–0.02)	0.06 (0.03–0.09)	0.02	4.09	* <0.001

* The mean difference is significant at the 0.05 level.

**Table 6:** Gaussian mixed-effects model statistics.

Outcome	Wald χ^2^ (df)	ICC	Marginal R^2^	Conditional R^2^	Athlete Variance	Residual Variance	Athlete SD	Residual SD
TD (m/min)	522.61 (1)	0.087	0.585	0.621	37.571	392.520	6.130	19.812
HID (m/min)	190.72 (1)	0.048	0.338	0.369	1.903	38.058	1.379	6.169
HID1 (m/min)	209.59 (1)	0.052	0.360	0.393	1.405	25.491	1.185	5.049
HID2 (m/min)	59.87 (1)	0.000	0.131	0.131	0.000	2.547	0.000	1.596
HIR (m/min)	63.43 (1)	0.001	0.138	0.139	0.003	2.887	0.057	1.699
HIR (nb/min)	82.73 (1)	0.030	0.179	0.204	0.001	0.017	0.023	0.130
Sprint/Dist (m/min)	0.00 (1)	NaN	NaN	NaN	0.000	0.000	0.000	0.000
Sprint/Nm (nb/min)	0.00 (1)	NaN	NaN	NaN	0.000	0.000	0.000	0.000
Zone3 (m/min)	390.86 (1)	0.070	0.513	0.547	3.300	43.731	1.817	6.613
Zone4 (m/min)	224.38 (1)	0.052	0.375	0.408	1.908	35.023	1.381	5.918
Zone5 (m/min)	63.43 (1)	0.001	0.138	0.139	0.003	2.887	0.057	1.699
Zone6 (m/min)	0.00 (1)	NaN	NaN	NaN	0.000	0.000	0.000	0.000
ACC2 (nb/min)	175.59 (1)	0.065	0.321	0.365	0.096	1.387	0.310	1.178
ACC3 (nb/min)	0.07 (1)	0.044	0.000	0.044	0.010	0.218	0.100	0.467
ACC4 (nb/min)	0.80 (1)	0.019	0.002	0.021	0.001	0.041	0.028	0.202
DEC2 (nb/min)	353.12 (1)	0.002	0.472	0.473	0.001	0.699	0.033	0.836
DEC3 (nb/min)	124.89 (1)	0.005	0.242	0.246	0.000	0.060	0.018	0.244
DEC4 (nb/min)	16.73 (1)	0.000	0.041	0.041	0.000	0.008	0.000	0.091

**Table 7:** Linear regression analysis of games with internal floaters and regular players.

Outcome Variable	Player Type	N	Mean ± Std	Predictor	Un-Standardized B	Sign	R^2^	F-Change	Constant	Regression Equation
TD (m/min)	Internal floater	18	140 ± 37.0	Relative pitch size	0.607	0.0695	0.191	3.78	43.9	y = 43.9 + 0.607x
	Regular player	248	137 ± 34.4	Relative pitch size	0.41	* <0.001	0.121	33.9	73.7	y = 73.7 + 0.41x
HID (m/min)	Internal floater	18	28.9 ± 22.6	Relative pitch size	0.497	0.0106	0.344	8.37	−50	y = −50 + 0.497x
	Regular player	248	37.4 ± 18.0	Relative pitch size	0.403	* <0.001	0.424	181	−25.1	y = −25.1 + 0.403x
HID1 (m/min)	Internal floater	18	24.7 ± 18.1	Relative pitch size	0.416	0.0067	0.378	9.71	−41.4	y = −41.4 + 0.416x
	Regular player	248	29.2 ± 15.0	Relative pitch size	0.303	* <0.001	0.346	130	−17.9	y = −17.9 + 0.303x
HID2 (m/min)	Internal floater	18	4.19 ± 6.90	Relative pitch size	0.0811	0.204	0.0986	1.75	−8.68	y = −8.68 + 0.0811x
	Regular player	248	7.05 ± 5.70	Relative pitch size	0.0855	* <0.001	0.19	57.5	−6.21	y = −6.21 + 0.0855x
HIR (m/min)	Internal floater	18	4.58 ± 7.30	Relative pitch size	0.0884	0.19	0.105	1.87	−9.46	y = −9.46 + 0.0884x
	Regular player	248	8.86 ± 7.60	Relative pitch size	0.106	* <0.001	0.168	49.6	−7.65	y = −7.65 + 0.106x
HIR (nb/min)	Internal floater	18	0.40 ± 0.50	Relative pitch size	0.00839	0.0691	0.192	3.8	−0.935	y = −0.935 + 0.00839x
	Regular player	248	0.72 ± 0.50	Relative pitch size	0.00727	* <0.001	0.213	66.4	−0.405	y = −0.405 + 0.00727x
Sprint/Dist (m/min)	Internal floater	18	0.00 ± 0.00	Relative pitch size						
	Regular player	248	1.19 ± 3.40	Relative pitch size	0.0143	* 0.050	0.0155	3.87	−1.02	y = −1.02 + 0.0143x
Sprint/Nm (nb/min)	Internal floater	18	0.00 ± 0.00	Relative pitch size						
	Regular player	248	0.08 ± 0.10	Relative pitch size	0.00115	* <0.001	0.0513	13.3	−0.0975	y = −0.0975 + 0.00115x
ACC2 (nb/min)	Internal floater	18	3.39 ± 2.40	Relative pitch size	0.0172	0.441	0.0375	0.624	0.663	y = 0.663 + 0.0172x
	Regular player	248	4.75 ± 2.00	Relative pitch size	−0.0132	* 0.0025	0.0365	9.33	6.8	y = 6.8 − 0.0132x
ACC3 (nb/min)	Internal floater	18	0.39 ± 0.50	Relative pitch size	0.00195	0.667	0.0118	0.192	0.076	y = 0.076 + 0.00195x
	Regular player	248	0.55 ± 0.50	Relative pitch size	−0.00285	0.0145	0.024	6.06	0.992	y = 0.992 − 0.00285x
ACC4 (nb/min)	Internal floater	18	0.07 ± 0.20	Relative pitch size	−0.0000343	0.986	0.00002	0.000298	0.0746	y = 0.0746 − 0.0000343x
	Regular player	248	0.04 ± 0.10	Relative pitch size	0.000153	0.509	0.00178	0.438	0.0136	y = 0.0136 + 0.000153x
DEC2 (nb/min)	Internal floater	18	3.38 ± 1.00	Relative pitch size	0.00754	0.423	0.0406	0.677	2.18	y = 2.18 + 0.00754x
	Regular player	248	4.09 ± 1.40	Relative pitch size	−0.00694	* 0.0201	0.0218	5.48	5.16	y = 5.16 − 0.00694x
DEC3 (nb/min)	Internal floater	18	0.67 ± 0.40	Relative pitch size	0.00334	0.393	0.046	0.772	0.142	y = 0.142 + 0.00334x
	Regular player	248	0.94 ± 0.60	Relative pitch size	0.00142	0.288	0.0046	1.13	0.721	y = 0.721 + 0.00142x
DEC4 (nb/min)	Internal floater	18	0.11 ± 0.20	Relative pitch size	0.00227	0.198	0.101	1.8	−0.249	y = −0.249 + 0.00227x
	Regular player	248	0.22 ± 0.30	Relative pitch size	0.00148	* 0.0222	0.0211	5.3	−0.0156	y = −0.0156 + 0.00148x
Zone3 (m/min)	Internal floater	18	40.3 ± 18.9	Relative pitch size	0.296	0.0843	0.175	3.39	−6.68	y = −6.68 + 0.296x
	Regular player	248	35.5 ± 12.2	Relative pitch size	0.182	* <0.001	0.188	57.1	7.29	y = 7.29 + 0.182x
Zone4 (m/min)	Internal floater	18	30.0 ± 20.6	Relative pitch size	0.487	* 0.005	0.398	10.6	−47.3	y = −47.3 + 0.487x
	Regular player	248	33.6 ± 16.6	Relative pitch size	0.336	* <0.001	0.349	132	−18.5	y = −18.5 + 0.336x
Zone 5 (m/min)	Internal floater	18	4.58 ± 7.30	Relative pitch size	0.0884	0.19	0.105	1.8	−9.46	y = −9.46 + 0.0884x
	Regular player	248	7.74 ± 6.10	Relative pitch size	0.0932	* <0.001	0.2	61.6	−6.72	y = −6.72 + 0.0932x
Zone6 (m/min)	Internal floater	18	0.00 ± 0.00	Relative pitch size						
	Regular player	248	0.05 ± 0.20	Relative pitch size	0.000306	0.434	0.00249	0.614	0.000599	y = 0.000599 + 0.000306x

* The mean difference is significant at the 0.05 level.

**Table 8:** Linear regression analysis of games with external floaters and regular players.

**Outcome Variable**	**Player Type**	**N**	**Mean ± Std**	**Predictor**	**Un-Standardized B**	**Sign**	**R^2^**	**F-Change**	**Constant**	**Regression Equation**
TD (m/min)	External floater	269	25.9 ± 17.4	Relative pitch size	0.072	0.0392	0.0158	4.29	18.5	y = 18.5 + 0.072x
	Regular player	491	128 ± 31.0	Relative pitch size	0.486	* <0.001	0.266	178	77.1	y = 77.1 + 0.486x
HID (m/min)	External floater	269	0.30 ± 0.90	Relative pitch size	0.00925	* <0.001	0.0928	27.3	−0.652	y = −0.652 + 0.00925x
	Regular player	491	26.1 ± 19.0	Relative pitch size	0.213	* <0.001	0.136	77	3.89	y = 3.89 + 0.213x
HID1 (m/min)	External floater	269	0.27 ± 0.80	Relative pitch size	0.00816	* <0.001	0.0923	27.1	−0.567	y = −0.567 + 0.00816x
	Regular player	491	20.5 ± 14.7	Relative pitch size	0.153	* <0.001	0.117	64.9	4.57	y = 4.57 + 0.153x
HID2 (m/min)	External floater	269	0.03 ± 0.20	Relative pitch size	0.00109	* 0.0097	0.0248	6.8	−0.0853	y = −0.0853 + 0.00109x
	Regular player	491	4.87 ± 6.00	Relative pitch size	0.0462	* <0.001	0.0652	34.1	0.0387	y = 0.0387 + 0.0462x
HIR (m/min)	External floater	269	0.03 ± 0.20	Relative pitch size	0.0012	* 0.0102	0.0245	6.69	−0.0915	y = −0.0915 + 0.0012x
	Regular player	491	6.04 ± 8.80	Relative pitch size	0.0643	* <0.001	0.0574	29.8	−0.673	y = −0.673 + 0.0643x
HIR (nb/min)	External floater	269	0.01 ± 0.00	Relative pitch size	0.000256	* 0.0086	0.0256	7.01	−0.0185	y = −0.0185 + 0.000256x
	Regular player	491	0.55 ± 0.60	Relative pitch size	0.00515	* <0.001	0.081	43.1	0.00916	y = 0.00916 + 0.00515x
Sprint/Dist (m/min)	External floater	269	0.00 ± 0.00	Relative pitch size						
	Regular player	491	0.74 ± 4.50	Relative pitch size	0.0142	*0.0218	0.0107	5.29	−0.736	y = −0.736 + 0.0142x
Sprint/Nm (nb/min)	External floater	269	0.00 ± 0.00	Relative pitch size						
	Regular player	491	0.06 ± 0.20	Relative pitch size	0.000703	* 0.0015	0.0205	10.2	−0.0179	y = −0.0179 + 0.000703x
ACC2 (nb/min)	External floater	269	0.92 ± 0.90	Relative pitch size	−0.00136	0.468	0.00197	0.527	1.05	y = 1.05 − 0.00136x
	Regular player	491	5.32 ± 2.60	Relative pitch size	−0.013	* <0.001	0.0273	13.7	6.68	y = 6.68 − 0.013x
ACC3 (nb/min)	External floater	269	0.32 ± 0.50	Relative pitch size	0.000411	0.66	0.00073	0.194	0.277	y = 0.277 + 0.000411x
	Regular player	491	0.81 ± 0.80	Relative pitch size	−0.00491	* <0.001	0.0365	18.5	1.32	y = 1.32 − 0.00491x
ACC4 (nb/min)	External floater	269	0.08 ± 0.20	Relative pitch size	−0.000289	0.457	0.00207	0.555	0.108	y = 0.108 − 0.000289x
	Regular player	491	0.06 ± 0.20	Relative pitch size	−0.000842	* <0.001	0.0233	11.7	0.152	y = 0.152 − 0.000842x
DEC2 (nb/min)	External floater	269	0.36 ± 0.70	Relative pitch size	0.000898	0.551	0.00133	0.356	0.267	y = 0.267 + 0.000898x
	Regular player	491	4.64 ± 2.00	Relative pitch size	−0.00638	* 0.0224	0.0106	5.25	5.31	y = 5.31 − 0.00638x
DEC3 (nb/min)	External floater	269	0.05 ± 0.20	Relative pitch size	0.000512	0.122	0.00893	2.41	−0.0000246	y = −0.0000246 + 0.000512x
	Regular player	491	1.12 ± 0.90	Relative pitch size	−0.00202	0.089	0.0059	2.9	1.33	y = 1.33 − 0.00202x
DEC4 (nb/min)	External floater	269	0.01 ± 0.10	Relative pitch size	0.000116	0.323	0.00366	0.98	−0.00258	y = −0.00258 + 0.000116x
	Regular player	491	0.24 ± 0.40	Relative pitch size	0.0000661	0.895	0.00004	0.0173	0.232	y = 0.232 + 0.0000661x
Zone3 (m/min)	External floater	269	0.98 ± 2.40	Relative pitch size	0.0172	* <0.001	0.0487	13.7	−0.784	y = −0.784 + 0.0172x
	Regular player	491	29.6 ± 14.0	Relative pitch size	0.149	* <0.001	0.122	67.8	14	y = 14 + 0.149x
Zone4 (m/min)	External floater	269	0.34 ± 1.00	Relative pitch size	0.0102	* <0.001	0.104	31.1	−0.707	y = −0.707 + 0.0102x
	Regular player	491	24.0 ± 16.4	Relative pitch size	0.175	* <0.001	0.123	68.4	5.7	y = 5.7 + 0.175x
Zone5 (m/min)	External floater	269	0.03 ± 0.20	Relative pitch size	0.0012	* 0.0102	0.0245	6.69	−0.0915	y = −0.0915 + 0.0012x
	Regular player	491	5.36 ± 6.40	Relative pitch size	0.0508	* <0.001	0.0685	36	0.0596	y = 0.0596 + 0.0508x
Zone6 (m/min)	External floater	269	0.00 ± 0.00	Relative pitch size						
	Regular player	491	0.01 ± 0.10	Relative pitch size	0.000276	* 0.0167	0.0117	5.77	−0.0161	y = −0.0161 + 0.000276x

* The mean difference is significant at the 0.05 level.

**Table 9:** Linear regression analysis of games with internal and external floaters and regular players.

Outcome Variable	Player Type	N	Mean ± Std	Predictor	Un-Standardized B	Sign	R^2^	F-Change	Constant	Regression Equation
TD (m/min)	Internal floater	21	88.0 ± 21.6	Relative pitch size	0.246	0.07	0.163	3.69	66.3	y = 66.3 + 0.246x
	External floater	89	34.1 ± 16.5	Relative pitch size	0.0463	0.354	0.0099	0.87	30.1	y = 30.1 + 0.0463x
	Regular player	206	98.7 ± 28.8	Relative pitch size	0.598	**<0.001**	0.538	237	45.4	y = 45.4 + 0.598x
HID (m/min)	Internal floater	21	3.83 ± 5.90	Relative pitch size	0.0903	**0.0106**	0.297	8.04	−4.12	y = −4.12 + 0.0903x
	External floater	89	0.41 ± 1.20	Relative pitch size	0.0119	**<0.001**	0.124	12.3	−0.603	y= −0.603 + 0.0119x
	Regular player	206	13.5 ± 13.4	Relative pitch size	0.309	**<0.001**	0.665	405	−14	y = −14 + 0.309x
HID1 (m/min)	Internal floater	21	3.49 ± 4.70	Relative pitch size	0.0773	**0.006**	0.335	9.57	−3.31	y = −3.31 + 0.0773x
	External floater	89	0.39 ± 1.10	Relative pitch size	0.0111	**<0.001**	0.129	12.9	−0.565	y = −0.565 + 0.0111x
	Regular player	206	11.1 ± 10.8	Relative pitch size	0.24	**<0.001**	0.619	331	−10.2	y = −10.2 + 0.24x
HID2 (m/min)	Internal floater	21	0.33 ± 1.30	Relative pitch size	0.0124	0.149	0.106	2.26	−0.763	y = −0.763 + 0.0124x
	External floater	89	0.03 ± 0.30	Relative pitch size	0.000772	0.334	0.0107	0.942	−0.038	y = −0.038 + 0.000772x
	Regular player	206	2.20 ± 3.50	Relative pitch size	0.0647	**<0.001**	0.435	157	−3.55	y = −3.55 + 0.0647x
HIR (m/min)	Internal floater	21	0.38 ± 1.50	Relative pitch size	0.0143	0.136	0.113	2.42	−0.883	y = −0.883 + 0.0143x
	External floater	89	0.03 ± 0.30	Relative pitch size	0.000849	0.334	0.0107	0.942	−0.0418	y = −0.0418 + 0.000849x
	Regular player	206	2.57 ± 4.00	Relative pitch size	0.0747	* <0.001	0.435	157	−4.08	y = −4.08 + 0.0747x
HIR (nb/min)	Regular player	21	0.05 ± 0.20	Relative pitch size	0.00193	0.0731	0.159	3.6	−0.119	y = −0.119 + 0.00193x
	External floater	89	0.00 ± 0.00	Relative pitch size	0.0000772	0.334	0.0107	0.942	−0.0038	y = −0.0038 + 0.0000772x
	Regular player	206	0.29 ± 0.40	Relative pitch size	0.00763	* <0.001	0.425	151	−0.391	y = −0.391 + 0.00763x
Sprint/Dist (m/min)	Internal floater	21	0.00 ± 0.00	Relative pitch size						
	External floater	89	0.00 ± 0.00	Relative pitch size						
	Regular player	206	0.15 ± 0.60	Relative pitch size	0.00393	* 0.002	0.048	10.3	−0.199	y = −0.199 + 0.00393x
Sprint/Nm (nb/min)	Internal floater	21	0.00 ± 0.00	Relative pitch size						
	External floater	89	0.00 ± 0.00	Relative pitch size						
	Regular player	206	0.02 ± 0.10	Relative pitch size	0.000608	* 0.002	0.0469	10	−0.0313	y = −0.0313 + 0.000608x
ACC2 (nb/min)	Internal floater	21	4.11 ± 2.40	Relative pitch size	0.0197	0.198	0.0856	1.78	2.38	y = 2.38 + 0.0197x
	External floater	89	1.52 ± 1.20	Relative pitch size	0.00433	0.211	0.0179	1.59	1.15	y = 1.15 + 0.00433x
	Regular player	206	5.65 ± 2.30	Relative pitch size	0.0032	0.486	0.00238	0.487	5.37	y = 5.37 + 0.0032x
ACC3 (nb/min)	Internal floater	21	0.32 ± 0.50	Relative pitch size	0.0022	0.469	0.0279	0.545	0.122	y = 0.122 + 0.0022x
	External floater	89	0.45 ± 0.50	Relative pitch size	0.00311	*0.043	0.0461	4.21	0.184	y = 0.184 + 0.00311x
	Regular player	206	0.88 ± 0.80	Relative pitch size	0.00102	0.535	0.00189	0.387	0.785	y = 0.785 + 0.00102x
ACC4 (nb/min)	Internal floater	21	0.06 ± 0.10	Relative pitch size	0.00054	0.567	0.0175	0.339	0.00742	y = 0.00742 + 0.00054x
	External floater	89	0.15 ± 0.20	Relative pitch size	0.00117	0.103	0.0302	2.71	0.0491	y = 0.0491 + 0.00117x
	Regular player	206	0.12 ± 0.30	Relative pitch size	−0.00076	0.125	0.0115	2.37	0.184	y = 0.184 − 0.00076x
DEC2 (nb/min)	Internal floater	21	2.83 ± 1.50	Relative pitch size	0.0149	0.127	0.118	2.55	1.52	y = 1.52 + 0.0149x
	External floater	89	0.64 ± 0.80	Relative pitch size	0.00214	0.372	0.00918	0.806	0.458	y = 0.458 + 0.00214x
	Regular player	206	4.36 ± 1.60	Relative pitch size	0.00544	0.084	0.0146	3.02	3.88	y = 3.88 + 0.00544x
DEC3 (nb/min)	Internal floater	21	0.42 ± 0.40	Relative pitch size	0.00546	* 0.019	0.259	6.64	−0.0593	y = −0.0593 + 0.00546x
	External floater	89	0.14 ± 0.30	Relative pitch size	0.000719	0.468	0.0061	0.53	0.0806	y = 0.0806 + 0.000719x
	Regular player	206	1.13 ± 0.80	Relative pitch size	0.00168	0.268	0.006	1.23	0.979	y = 0.979 + 0.00168x
DEC4 (nb/min)	Internal floater	21	0.05 ± 0.20	Relative pitch size	0.00162	0.120	0.122	2.65	−0.0967	y = −0.0967 + 0.00162x
	External floater	89	0.03 ± 0.10	Relative pitch size	0.000018	0.959	0.00003	0.00269	0.0236	y = 0.0236 + 0.000018x
	Regular player	206	0.22 ± 0.30	Relative pitch size	0.00113	0.084	0.0146	3.02	0.12	y = 0.12 + 0.00113x
Zone3 (m/min)	Internal floater	21	12.4 ± 11.6	Relative pitch size	0.09	0.229	0.0751	1.54	4.5	y = 4.5 + 0.09x
	External floater	89	1.19 ± 2.70	Relative pitch size	0.0168	* 0.037	0.0491	4.49	−0.246	y = −0.246 + 0.0168x
	Regular player	206	20.4 ± 12.6	Relative pitch size	0.233	* <0.001	0.425	151	−0.319	y = −0.319 + 0.233x
Zone4 (m/min)	Internal floater	21	4.31 ± 5.50	Relative pitch size	0.0909	* 0.006	0.34	9.81	−3.69	y = −3.69 + 0.0909x
	External floater	89	0.48 ± 1.30	Relative pitch size	0.013	* <0.001	0.123	12.2	−0.634	y = −0.634 + 0.013x
	Regular player	206	13.4 ± 12.1	Relative pitch size	0.273	* <0.001	0.633	353	−10.9	y = −10.9 + 0.273x
Zone5 (m/min)	Internal floater	21	0.38 ± 1.50	Relative pitch size	0.0143	0.136	0.113	2.42	−0.883	y = −0.883 + 0.0143x
	External floater	89	0.03 ± 0.30	Relative pitch size	0.000849	0.334	0.0107	0.942	−0.0418	y = −0.0418 + 0.000849x
	Regular player	206	2.44 ± 3.80	Relative pitch size	0.0713	* <0.001	0.451	167	−3.9	y = −3.9 + 0.0713x
Zone6 (m/min)	Internal floater	21	0.00 ± 0.00	Relative pitch size						
	External floater	88	0.00 ± 0.00	Relative pitch size						
	Regular player	206	0.00 ± 0.00	Relative pitch size	0.000123	* 0.003	0.042	8.94	−0.00614	y = −0.00614 + 0.000123x

* The mean difference is significant at the 0.05 level.

## Data Availability

Data might be provided upon reasonable request.

## Abbreviations

The following abbreviations are used in this manuscript:

SSG	Small-Sided Games
TD/min	Relative Total Distance
HIR	High-Intensity Running
Sprint/Nm	Number of Sprints
Sprint/Dist	Sprint Distance
ACC	Acceleration
DEC	Deceleration
RTP	Return-to-Play
GPS	Global-Positioning units
CI	Confidence Intervals
CL	Confidence Limits
Effect sizes	ESs
